# Exploring diagnosis and treatment of premenstrual dysphoric disorder in the U.S. healthcare system: a qualitative investigation

**DOI:** 10.1186/s12905-023-02334-y

**Published:** 2023-05-17

**Authors:** Kiera Chan, Anna A. Rubtsova, Cari Jo Clark

**Affiliations:** Emory University, Atlanta, United States

**Keywords:** Premenstrual Disorder, Qualitative research, Treatment, Diagnosis, Menstrual Health, Healthcare Research

## Abstract

**Background:**

Premenstrual Dysphoric Disorder (PMDD) is a premenstrual condition that affects 3–8% of the US population, yet knowledge on treatment and consistent diagnostic testing is lacking. While research concerning the epidemiology and pharmaceutical treatments for this condition has increased, there is a lack of qualitative studies on the experiences of patients who live with this condition. The aim of this study was to explore the diagnostic and treatment experiences of PMDD patients in the U.S. healthcare system and identify barriers to diagnosis and treatment.

**Methods:**

This study uses a feminist framework with qualitative phenomenological methods. We recruited participants who identified as having PMDD, regardless of official diagnosis, through online forums within the U.S. PMDD community. The study conducted 32 in depth interviews with participants on their experiences with PMDD diagnosis and treatment. Thematic analysis methods revealed key barriers within the diagnostic and care process including patient, provider, and societal barriers.

**Results:**

This study presents a PMDD Care Continuum that represents the timeline of participant experiences beginning from symptom onset towards official diagnosis, treatments, and ongoing management of the condition. Participant experiences demonstrated that much of the diagnostic and treatment processes were burdened on the patient, and that successful navigation within the healthcare system was dependent on high levels of self-advocacy.

**Conclusions:**

This was the first study to describe the qualitative experiences of patients who identified as having PMDD in the U.S. Further research is needed to refine and operationalize diagnostic criteria and treatment guidelines for PMDD.

**Supplementary Information:**

The online version contains supplementary material available at 10.1186/s12905-023-02334-y.

## Introduction

Premenstrual Dysphoric Disorder (PMDD) is a condition that affects 3–8% of menstruators, yet knowledge on treatment and consistent diagnostic testing is lacking [1, 2]. PMDD is defined as a “cyclical recurrence of distressing or impairing affective symptoms,” which must appear 3–4 days before menstrual bleeding [3]. PMDD is characterized by an abnormal response in the brain towards a normal monthly change in hormone levels post ovulation [3]. The Global Burden of Disease Study (GBD) estimated PMDD to be 0.5 on a scale from “0” (perfect health) to “1.0” (death) of health loss from disease, pointing to public health relevance [4]. Furthermore, both the incidence of PMDD and related suicide reports are increasing globally [5]. Yet, only recently this condition started gaining recognition from medical professionals. In 2013, PMDD was included in the DSM-V as a distinct category, which lead to its subsequent inclusion in the 11th edition of World Health Organization’s (WHO) International Classification of Disease (ICD) [6–8]. Despite these advancements, there are issues with PMDD diagnosis and treatment noted in the current literature [1, 2].

There are disparities in diagnostic tools and methods for PMDD, creating issues for diagnosis and proper treatment [9, 10]. Within the DSM itself, the criteria have been evaluated as ambiguous, leaving most of the responsibility on the clinician’s ability to accurately assess and diagnose [11]. There is no universal standardized diagnostic measuring tool assessing for PMDD [12]. Additionally, due to the lack of understanding of the etiology of PMDD, lack of ability to test for biomarkers for PMDD, and the complex nature of the behavioral and affective symptoms, there are limited options for treatment [13].

It takes an average of 20 years for women to be accurately diagnosed and treated for PMDD [14]. However, there may be significant health benefits for women to receive timely diagnosis as women who are diagnosed later in life are more likely to attempt suicide [14]. Individuals with undiagnosed PMDD report impairment in work productivity, lost wages, and higher medical expenses [15, 16]. In general, women have been known to be mistreated in the U.S. healthcare system, waiting longer for medical attention or having their pain dismissed more than men [17–20]. Currently, only one qualitative study examined patient experiences with PMDD using a sample from the United Kingdom, suggesting a need for further research, particularly within the United States [14].

To develop an understanding of PMDD from the patient’s perspective, this study uses approaches from feminist phenomenology to explore the lived experiences of individuals who identify as having PMDD [21, 22]. Using a feminist perspective to gain understanding of this condition will disrupt the traditional androcentric biomedical discourse on PMDD. The aim of this study is to identify barriers to diagnosis and successful treatment of PMDD in the US healthcare system. This research is to our knowledge unique, as it describes the lived experiences of patients with PMDD in the US. The results of this study will help to address the knowledge gap around PMDD by providing a qualitative sample of patients’ perspectives on their experiences within the US healthcare system.

## Materials and methods

### Participants and sample

The study context for this project was chosen as the United States patient population of PMDD. The eligibility criteria included: (1) anyone who identified as having PMDD, regardless of official diagnosis; (2) able and willing to provide informed consent; (3) able to complete interview in English language; (4) US resident aged 18 years and older. Participants were recruited in partnership with the Non-Profit, International Association for Premenstrual Disorders (IAPMD) via online advertisements. Snowball sampling was also used to recruit participants through current participants and Non-Profit members. A total of 32 participants were recruited in the study in July 2021.

### Data collection

Each confidential interview lasted about one hour and was conducted over Zoom or phone call. Participants were asked a series of questions pertaining to their experiences with PMDD, their process of self-diagnosis or provider diagnosis (if relevant), their past and current methods of treatment, and their experiences in the US healthcare system. The interviews followed a semi-structured interview guide consisting of 25 questions (See Appendix A). Prior to the interview, oral consent was received. The interviews were recorded over Zoom, transcribed, and de-identified by trained study members.

### Data analysis

This study used a feminist phenomenological approach to explore the themes generated from the in-depth interviews to understand the lived experiences of patients with PMDD. By allowing participants to subjectively define whether they identified as having PMDD, phenomenology was used to understand the perspective of the patient within the healthcare system. A feminist framework intends to understand a phenomenon through the eyes of marginalized groups, as it recognizes that marginalized locations are epistemically superior in that they can deconstruct privilege and unveil previously unknown facts [22].

A codebook was created using inductive and deductive methods (See Appendix B). A total of 6 coders split the 32 transcripts evenly and coded simultaneously in MaxQDA software. Intercoder reliability was assessed through a comparison of coded transcripts from two independent and blinded coders. Intercoder reliability scores were not calculated. The broad themes that emerged from the conceptual model were confirmed by constantly going back to participant narratives, repeating data searches, and reviewing codes. Analysis consisted of coding data by inductive and deductive themes, conducting structured comparisons across the sample, developing thick descriptions, categorizing themes, and conducting case and code-based analysis. From this analytic process, a conceptual model was created using the experiences of the participants, building upon a model derived deductively from a tuberculosis care continuum [23]. This PMDD Care Continuum is characterized by overlapping barriers that cause specific delays in the patient’s care continuum as well as certain feedback loops as patients move through the healthcare system.

### Ethical considerations

Ethical review was exempted by the Emory University IRB, because it was deemed that it met the criteria for exemption under 45 CFR 46.104(d)(2). All data was deidentified prior to analysis. Oral Informed consent was obtained from all subjects and it was approved by Emory IRB. Prior to data collection, all portions of the study materials were reviewed by the Emory IRB (ID number 00002906). The exemption was determined on July 23rd, 2021. All methods were carried out in accordance with relevant guidelines and regulations or declaration of Helsinki. Oral consent was obtained from all subjects and it is approved by Emory IRB.

## Results

### Sample description

As shown in Table 1, there were a wide range of demographic characteristics throughout the sample. Although the sample was not diverse in terms of race, there was a wide range of diversity in terms of household income. The average years from symptom onset to official diagnosis was 5.6 years. Overall, most participants (87%) in the sample had received an official diagnosis. The sample was highly educated, with over half having completed some college. The average age was 33.9 years, with the youngest participant being 21 years old and the oldest participant 50 years old. Over half of the sample was single. Most participants were employed. Participants suffered PMDD for a mean of 17.43 years and experienced a mean of 5.56 years from symptom onset to official diagnosis.

**Table 1:** n = 32

Variable	Age Range	M(SD) or %
Gender		
Female		100%
Male		0%
Age	29 years [21–50]	33.9 (7.99)
Employed		71%
Race		
White		94%
Mixed		3%
Alaskan Native		3%
Hispanic		12%
Education Level		
Completed High School		6%
Some college (undergraduate)		19%
Bachelor’s degree		45%
Graduate school in progress		6%
Master’s degree		13%
Beyond Graduate school		3%
Marital Status		
Single		65%
Married		29%
Divorced		6%
Household Income		
<=25,000		32%
> 25,000 and < = 50,000		32%
> 50,000 and < = 75,000		6%
> 75,000 and < = 100,000		6%
> 100,000		23%
Comorbidities		
Total comorbidities		59%
Women’s health conditions		37%
Endometriosis		19%
Autoimmune conditions		6%
How many years suffered from PMDD		17.43 (8.86)
Official PMDD Diagnosis		87%
Years since official diagnosis		5.56 (5.63)

**Note:** “unemployed” includes students

### PMDD symptoms/ experiences

There were a range of experiences of PMDD across the sample. Most patients suffered onset of symptoms during their first menses. The youngest patient was officially diagnosed at age 16 and the oldest was at age 45. Five participants described being sent to a psychiatric institution specifically for PMDD. A few participants described suicide attempts as a result of worsening PMDD symptoms. Almost all 32 participants experienced suicidal ideation as a part of their monthly PMDD symptomology. Many participants were prescribed around 5 different medications for PMDD. Although not specifically asked in the interview guide, many participants brought up that they were single, unemployed, or even lacked housing due to PMDD. Others mentioned that they were unable to complete their schooling or pursue the type of career they aspired to as a result of their condition impairment.

Patient Delay.

**Fig. 1:**
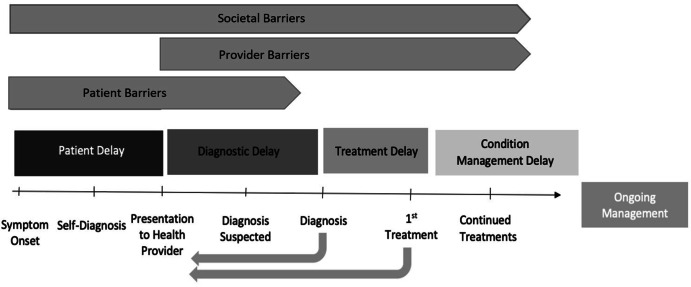
The PMDD Care Continuum captures the common pathways to diagnosis and treatment among participants. Below, we will describe our findings in relation to the elements of the model, including Patient Delay, Diagnostic Delay, Treatment Delay, and Condition Management, paying attention to various barriers connected to delays in care. The barriers are defined by who experiences them in the continuum

“Patient delay” describes the portion of the PMDD Care Continuum from symptom onset to presentation of PMDD to the patient’s health provider. Many patients presented their symptoms to providers, but providers failed to recognize PMDD symptoms and would tell patients that it was “in their head.” As shown in Fig. 1, *societal barriers* impacted patient delay, specifically towards patients’ self-perception of what was normal and what was considered abnormal menstrual symptoms. Participants stated that this internalization of the normalization of menstrual suffering delayed them from seeking medical care. *Patient barriers* were barriers that limited the individual’s ability to seek care such as not talking about their symptoms to others, not speaking against a doctor if misdiagnosed, or experiencing access barriers to healthcare. As shown in Fig. 1, *Patient Barriers* are impacted by *Societal barriers* such as menstrual stigma limiting women from discussing their symptoms or the expectation that women should be selfless and take care of others, encouraging them to suffer in silence.

**Experiencing Misdiagnosis.** Overall, nine participants experienced misdiagnoses, five of whom experienced later PMDD self-diagnosis, and the rest were diagnosed officially with PMDD later by a provider. Most participants were misdiagnosed with bipolar or borderline personality disorder. Other misdiagnoses included schizophrenia, chronic fatigue, fibromyalgia, Post Traumatic Stress Disorder, and Cluster B Personality Disorder. Some patients were so desperate for answers and treatment that they accepted their misdiagnosis, even if it did not necessarily fit them. One patient described why she might have been misdiagnosed:

They explored bipolar, but I didn’t meet the bipolar diagnosis because I didn’t have the mania. So, I didn’t have that. I didn’t have the major depression because it wasn’t sustained. And so, it was just like... I never really fit into a good box, I guess. Which makes sense because the box wasn’t really established until 2017. *Sarah, Age 34, 21 Years PMDD Sufferer*

Participants suffered health consequences from being on the wrong medications for so long and struggling to get off these medications. As shown in Fig. 1, *patient barriers* also impacted patient’s ability to seek care. For instance, dismissal of symptoms, misdiagnosis, and other negative experiences led to medical trauma and mistrust of doctors, further preventing patients from seeking help.

**Self-Diagnosis**. 19 out of the 32 total participants self-diagnosed, 17 of whom later received official diagnoses from providers. Three other participants were diagnosed by a partner or parent (mother). Many patients experienced catalysts that caused them to realize that their symptoms were not normal, leading them to research and self-diagnose. Several patients talked to other women about their menstrual cycles and would realize that other women do not experience PMDD symptoms. Other patients would start or stop certain medications, which would either exacerbate or relieve their PMDD symptoms. For instance, starting or stopping certain hormonal birth controls caused this phenomenon, which led patients to realize that they were experiencing cycle related symptoms, which led them to research PMDD. Some patients self diagnosed through their attempts to have children. A few patients visited fertility doctors, which led them to identify their PMDD symptoms. A few other patients stopped their hormonal birth control to try to have children, which caused severe PMDD symptoms that had been previously alleviated by the birth control.

### Diagnostic delay

Very few patients had one doctor that followed them through their entire PMDD Care Continuum. Thus, many patients found a doctor to diagnose, but had to switch providers to receive treatment. Many patients, even after receiving an official diagnosis from a provider, could not bring this diagnosis with them to their next provider, as each provider had to reevaluate. Provider barriers impacted diagnosis in that PMDD was a relatively “new” condition having been recently added to the DSM, so participants reported that their providers did not think it was real. Other *Provider Barriers* occurred as patients often were dismissed or experienced medical gaslighting. Medical gaslighting refers to doctors normalizing or dismissing patients’ symptoms. Patients described medical gaslighting, as shown in the participant’s quote below, as impacting their ability to distinguish their symptoms as well as advocate for themselves in the healthcare system.

They have a- an issue saying, ‘I need to learn more about this-’. But instead -- instead they’ll say, ‘no you’re wrong because I don’t know about it.’ And that’s pretty gaslightly, you know. Medical gaslighting is a real thing, especially in Women’s Health. *Kai, Age 29, 12 Years PMDD Sufferer*

**Official Diagnosis.** Many patients described receiving an official diagnosis as a validating experience. Common pathways to diagnosis included women recognizing the cyclical nature of their symptoms, researching PMDD, and finding a doctor to diagnose. Some patients saw up to 10 different providers before receiving an official diagnosis, which took years. Overall, there were many *provider barriers* that limited providers’ ability to correctly diagnose PMDD (Fig. 1). Patients described the power dynamic between doctor and patient as a barrier, which resulted in many providers not listening to the patient. For instance, several patients tracked their cycles and symptoms and brought these documents to their provider, but the provider did not even look at these documents.

**Type of Provider.** As shown in Fig. 1, the *provider barriers* prevented providers themselves from being able to diagnose PMDD. Some participants stated that doctors did not have the tools or knowledge to diagnose or treat PMDD. Overall, patients in our study had the most negative experiences with gynecologists. Gynecologists tended to treat menstrual issues only related to physical abnormalities, such as uterine fibroids, so all other cycle related issues were dismissed. Participants discussed that General Practitioners were more likely to diagnose PMDD as PMS. Therapists were often described as supportive because they listened to the patient and were more likely to research PMDD.

**Female vs. Male Doctors.** Participants stated that they had different experiences with female and male providers. With female providers, participants said that they often lacked empathy, because female providers experienced menstruation as well. Normalizing women’s symptoms as something all women had to deal with was described by 9 participants. Overall, male doctors were described by participants more often in dominant characteristics, such as implying that they knew more about the patient’s experience than the patient. A total of 8 different participants described a male provider being disrespectful. For instance, one male provider described the participant as living “like a rat,” and a separate participant was told her uterus was “mad at her” for not having children. More participants described male providers as “clueless” regarding PMDD compared to female providers.

### Treatment delay

Many patients felt that they must prove to doctors how severe their symptoms were in order to be taken seriously. Some patients used prior hospitalizations in a psychiatric ward as proof or support for the severity of their condition. Others were hesitant to tell their provider about their suicidal ideation because they were afraid of being hospitalized or not taken seriously. Patients shared that they had to jump through many hoops in order to receive the type of treatment they wanted.

**Type of Treatment.** Participants had varying experiences with types of treatment they received for PMDD over the course of their PMDD care continuum. Three participants received chemical menopause treatments and five participants underwent surgical menopause in the sample. Many patients described some form of menopause, whether chemical or surgical as the ultimate, but unrealistic goal for treatment. 29 out of 32 participants reported being prescribed some type of antidepressants or SSRIs. Many patients felt that first line treatments did not directly address PMDD, because they experienced only partial symptomatic relief. One patient described these treatments as “sideways treatments.” Even though providers were correct in prescribing these treatments as per diagnostic guidelines, their actions were perceived by participants to mean that they were “merely depressed,” because it was an off-label use (providers used SSRIs to treat a menstrual health condition, but SSRIs are designed to treat depression). Patients felt that they were not listened to by their providers, because they would convey that they had already been on these treatments before with little or no relief, but providers would still prescribe them and insist that these were their only options. Some participants had negative side effects from both SSRIs and hormonal contraceptives but were not offered any other options to manage their PMDD, because providers only used first line treatments to treat PMDD.

Per participants’ perceptions, providers felt it was acceptable or normal for women to experience some degree of menstrual pain and suffering, because first line treatments only gave partial relief but were easily prescribed. Most patients who were treated with non-first line treatments felt complete relief of their symptoms and described higher quality of life compared to their experience on the first line treatments. Participants expressed that first line treatments only “managed” their symptoms during one part of their menstrual cycle, whereas participants who used second-, third-line, or alternative non-traditional treatments reported experiencing more functionality or no PMDD symptoms at all. Furthermore, participants reported uneasiness around providers’ practice of prescribing first line treatments, because providers prescribed without following up. If there was a follow up, it was often 9 weeks later, which participants mentioned was enough time for them to feel suicidal after experiencing negative side effects from the medication. Providers did not proceed with caution with first line treatments as they did with other second or third line treatments. For instance, they had strong hesitations or refused to treat patients with hormonal or surgical menopause but had no hesitation in prescribing birth control or SSRIs. No participant mentioned having suicidal ideation as a side effect of surgical or chemical menopause, yet this was a common side effect of being put on the “wrong” type of SSRI or hormonal contraceptive.

**Co-morbid Conditions.** In some cases, having another condition helped patients receive treatment. For instance, one patient had a disability in conjunction with PMDD, so having a large care team for their disability helped in getting treatment for PMDD. The participant’s PMDD symptoms were taken more seriously because they had a disability that could be impacted by the PMDD symptoms. We found that *societal barriers* played a role at this stage as well (see Fig. 1). Participants described society putting emphasis on women’s fertility and since PMDD does not impact women’s fertility, it is a condition that is often left untreated. Four out of the five participants who received surgical menopause had other female conditions that impacted fertility (e.g. endometriosis).

### Condition management delay

Even after years with an official PMDD diagnosis, patients still struggled to find a provider to treat them or find a successful treatment method. In Fig. 1, the feedback loops show that many patients repeat the process of finding a health provider and presenting their case after their official diagnosis or after having no success with their first treatment. This cycle continues until patients can find a provider to help manage their condition. A few participants received misdiagnoses even after their official PMDD diagnosis. Many participants described PMDD as a difficult and complex condition, with one participant stating:

It’s a woman’s issue which already gets tossed aside and it’s a mental health issue which also gets tossed aside. It’s just- it’s a double whammy of bad luck and they just don’t want to take it seriously for whatever reason. … They just wanna think it’s either brain issue or it’s a reproductive system issue and they can’t seem to connect that it’s- they’re both. They’re tied into each other. And that the body is making the brain feel this way. *Rachel, Age 29, 18 Years PMDD Sufferer*

Within the sample, most participants described being “lost” in the cycle of the healthcare system, being passed from one provider to the next, lacking continuity of care, and repeating the process of diagnosis and treatment with each new provider. Many providers quit on PMDD patients, stating that they could no longer help them. *Societal barriers* included PMDD being recently added to the DSM, which led providers to not “believe” in the condition. Without a clear designation of diagnosis, patients were forced to be responsible for themselves, as no one took responsibility for their condition and treatment. One participant described this phenomenon as the “bystander effect.” Other patients turned to alternative medicine if they lacked success in the traditional healthcare system or did not have access to health insurance. Two participants managed their condition by micro dosing with psilocybin, and a few others used other methods such as reiki, marijuana, acupuncture, and homoeopathic treatments. One participant claimed that she was “self-healed,” using nontraditional methods of therapy such as meditation, diet, supplements, and other holistic measures to manage her condition, without a doctor.

Most participants who were able to successfully manage PMDD still experienced a monthly “hell week” that interfered with daily functioning. Seven participants described lack of success with finding a method to manage their condition.

## Discussion

**Fig. 2:**
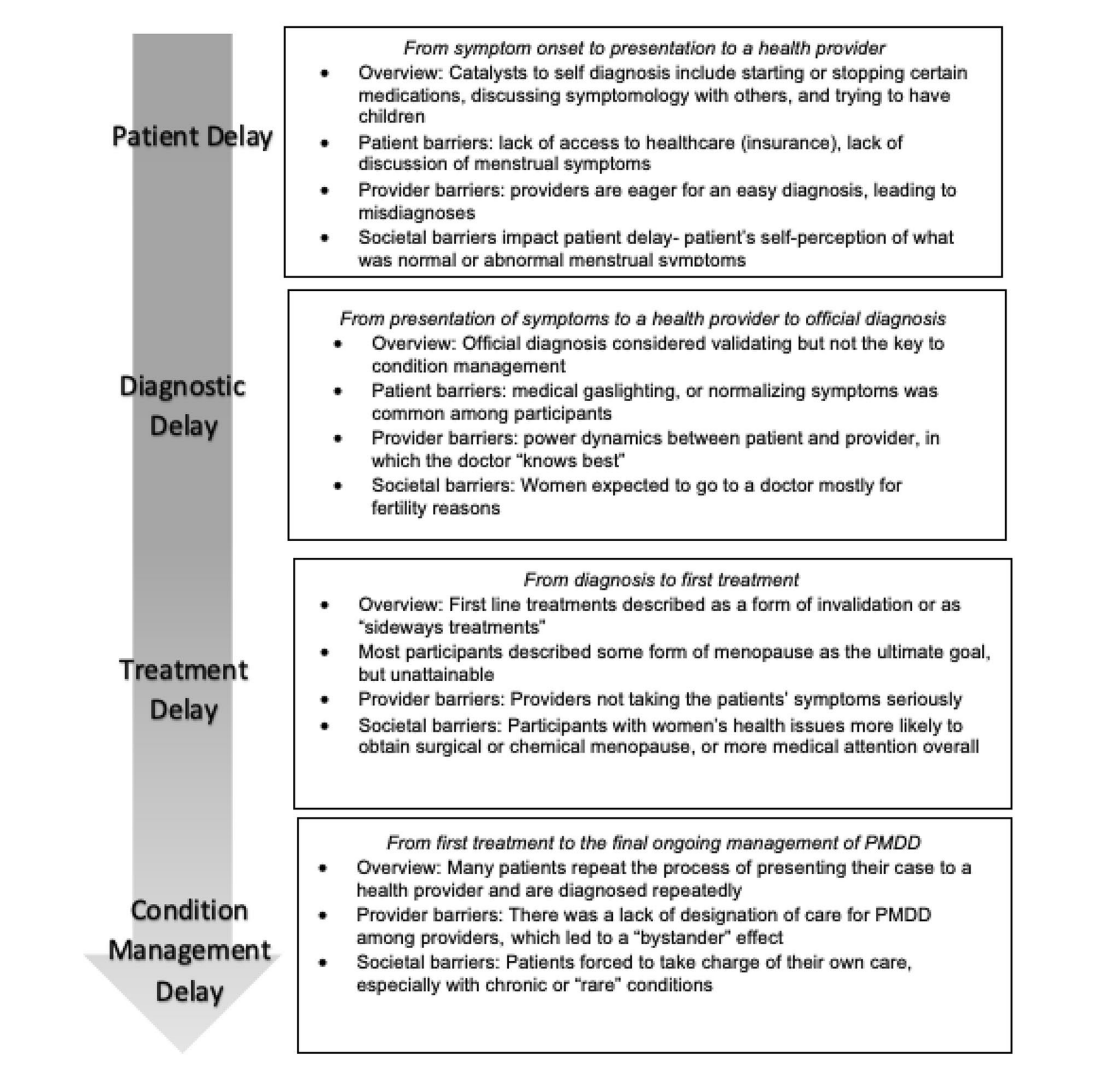
Care Continuum Delays

In this study, we report the healthcare experiences of patients with PMDD using a conceptual model that describes the delays to diagnosis and treatment. Overall, this study found that a multitude of societal, provider, and patient related barriers (as summarized in Fig. 2) were connected to delays in diagnosis and treatment, starting from symptom onset to condition management. The PMDD Care Continuum reported in the results reflected the cyclical nature of this process as patients became caught up in the healthcare system. The most compelling finding from this study was that an official diagnosis did not result in doctors being able to treat PMDD. Participants had to be reevaluated and diagnosed again with every new provider they saw. This was the first study of its nature to describe the qualitative experiences of patients who identified as having PMDD in the U.S.

One significant finding from this study was the phenomenon of “medical gaslighting.” This phenomenon has also been observed in other studies on premenstrual disorders in which provider minimize patients’ symptoms [23]. This study found that women had varying self-perceptions of Premenstrual Syndrome (PMS) creating problems to distinguish PMS from PMDD (Reilly, 1999). Other studies on premenstrual disorders, such as endometriosis or PCOS have also found common themes in the recurring dismissal of patient symptoms and lack of empathy by providers [23–26]. The findings on women’s diagnosis experiences in the UK by Osborn et al (2020) concerning the invalidation by providers and misdiagnosis were also confirmed by our study. Consistent with previous research on premenstrual disorders, this study also found that women were not taken seriously unless their symptoms impacted their fertility [27].

The finding that women are turning to alternative medicine as a result of unsuccessful prior Western medical treatments is particularly salient for providers as women are turning to methods in which they have more autonomy over their bodies and less negative side effects compared to first line PMDD treatments. Many patients did not have success on first line treatments due to many side effects, which correlates with findings that patients tend to have worse psychosocial outcomes on hormonal contraceptives [28]. Doctors tended to prescribe any type of hormonal contraceptive, with few patients being prescribed YAZ, the only FDA approved hormonal contraceptive for PMDD, suggesting a need for additional medical education among providers.

One unique finding from this study was that participants experienced negative outcomes with both male and female providers. Previous studies indicated that women receive better care from female doctors than males [29–31]. Our findings illustrated that patients had varying experiences depending on the type of provider they visited. Unlike Hantsoo et al.’s previous study on specialty providers and patient experiences with PMDD, our study found that patients reported worse experiences with gynaecologists [32]. However, our finding that patients had better experiences (e.g. more compassion and validation) with psychotherapists was supported by their study. Self-advocacy used to navigate the healthcare system was another significant finding confirmed by women’s health research [33–35], as most of the burden of diagnosis and treatment in our study rested on the patient.

This study has several strengths and limitations. Our results provide novel insight into how patient-practitioner communications act as barriers to diagnosis and management of PMDD. There were several limitations to this study. Our sample lacked diversity in race or socioeconomic status. There were difficulties in recruiting patients without an official PMDD diagnosis in order to reveal a diverse range of diagnostic experiences. Since socioeconomic status and race can impact the patient’s ability to access healthcare, it is crucial that future studies include more diverse samples of participants to capture the full understanding of barriers in the US healthcare system.

Several key points are salient to researchers and practitioners. First, participants described a lack of coordination among healthcare providers and specialties, which resulted in further misdiagnosis or patients having to repeat previously unsuccessful treatments. Studies have shown that a lack of coordination of care can result in serious medical complications for the patient [36–38]. We recommend that up-to-date PMDD resources be provided to healthcare workers to aid with diagnostic practices and early intervention or treatment. Although the data suggest that self-advocacy is a key part of the process of diagnosing and managing PMDD among patients, these strategies are limited as patients in our study struggled to gain a diagnosis or the preferred method of treatment. Thus, further research on the providers’ side of diagnosis and treatment is needed.

## Conclusion

This study concludes that patients with PMDD experience numerous barriers to diagnosis and treatment in the U.S. healthcare system. Our findings showed that patients with PMDD are often caught in a cycle of diagnostic and treatment delay within the US healthcare system. We recommend further research on the diagnostic practices of PMDD as well as gaining the perspective of healthcare providers in regard to diagnosis of PMDD. These findings contribute to the overall body of research on premenstrual disorders as well as diagnosis literature on women’s health conditions, because it presents a unique conceptual model on delays and barriers to condition management. These findings will provide the basis for further research on the operationalization and refining of diagnostic criteria for PMDD.

## Electronic supplementary material

Below is the link to the electronic supplementary material.

Additional File 1: The PMDD Care Continuum captures the common pathways to diagnosis and treatment among participants

## Data Availability

Supplemental files are included in the form of the interview codebook and interview guide. Raw data is not provided as it is too large (32 interview transcripts, each an hour long). However, the datasets used and analysed during the current study are available from the corresponding author on reasonable request.
