# Supplementary material for: Exploring diagnosis and treatment of premenstrual dysphoric disorder in the U.S. healthcare system: a qualitative investigation
Source: BMC Womens Health. 2023 May 17;23:272. doi: 10.1186/s12905-023-02334-y (PMC10193729; doi:10.1186/s12905-023-02334-y)
Supplement: Supplementary file 1 — Additional File 1: The PMDD Care Continuum captures the common pathways to diagnosis and treatment among participants [file 12905_2023_2334_MOESM1_ESM.docx]

**Appendix A: Interview Guide**

*Time: 1 hour* | 15-25 questions

**Interview on Experiences with Premenstrual Dysphoric Disorder**

Date:

Start Time:

Stop Time:

Participant study ID____________________

**INTRODUCTION AND CONSENT**

Hello,

Thank you for taking part in this interview. My name is…. I am a Master’s of Public Health student at Emory University working on my thesis project. For this study, I would like to learn about the experiences of individuals with Premenstrual Dysphoric Disorder. I am interested in learning about personal experiences, attitudes and opinions on diagnosis or treatment, and challenges within the healthcare system for people with PMDD or providers who treat PMDD. During this interview, you will be asked questions around personal or general experiences or attitudes on PMDD and how it is experienced within the healthcare system. Your responses may help inform the PMDD community on how to create more support services and potentially help others with PMDD or medical providers better understand how to treat individuals with PMDD. You are the expert here and I’m thankful that you are sharing your perspective with me. Do you have any initial questions for me?

[Perform oral consent *(See Informed Consent document)*.]

With all of this information, would you like to participate in the interview today?

Do I have your permission to record our discussion?

Are you ready to proceed?

------------------------------------------------------------------------------------------------------------

**Socio Demographic Questions**

1. What is your age?
2. What is your occupation?
3. What is your sex? What gender do you most identify with?
4. What is your race?
5. Are you of Hispanic origin?
6. What is your education?
7. What is your marital status?
8. What is an annual income before taxes of your household?
   1. <=25,000
   2. >25,000 and <=50,000
   3. >50,000 and <=75,000
   4. >75,000 and <=100,000
   5. >100,000
9. How long have you suffered from PMDD?__________________________
10. Have you received an official PMDD diagnosis? Yes/No    If yes, when?___________

**Initial Experiences with PMDD**

“So, let’s start off with you telling me a little about yourself and how you became involved in the PMDD community.”

1. When was the first time you ever heard about the condition, PMDD?
   1. How did you come to know about PMDD?
2. If you personally had experiences with PMDD, could you tell me a little about your first manifestations?
   1. What have been your main symptoms?
   2. How have these symptoms impacted your daily life?
   3. How have they impacted your relationships?
   4. How have they impacted your ability to work or function in society?
3. If you do not want to talk about personal experiences, what have you heard about other community members’ first experiences or how they came to know about PMDD?
4. What have been your experiences with the PMDD community so far?
   1. Have you been treated in the medical community?
   2. Have you been treated by alternative medical therapists?
   3. Have you participated in online events or any support groups?
   4. Have you helped to provide PMDD related services to others?

**Experiences With Diagnosis**

“Now I’d like to learn a little bit about how you or other people you may know have been diagnosed with PMDD.”

1. Earlier you said that you have (have not) received PMDD diagnosis.
   1. If so, would you mind explaining your experience with the diagnosis process?
   2. If you have not received the diagnosis, then why?
2. In your opinion, what is the usual experience with getting diagnosed with PMDD?
   1. Is the process easy? How so?
   2. Is the process hard? How so?
3. If you haven’t received a diagnosis or do not want to talk about your own experience, would you describe someone else’s experience with diagnosis?
   1. This could be a fellow PMDD member’s experience.
4. How did you feel/ how do people usually feel when you/ they receive a diagnosis?
   1. Would you describe this feeling?
   2. What kind of emotions are brought on with a new diagnosis with PMDD?
5. Does receiving a diagnosis change one’s experience with PMDD?
   1. How so?
   2. Prompts: changes in treatment of symptoms, changes in effects on daily life.
6. Have you ever had any problems with misdiagnosis?
   1. Have you heard of others experiencing misdiagnosis?
7. What do you think was something that helped you receive a diagnosis?
   1. Or- if you have not received a diagnosis, what would help you or others receive one?
8. What do you think hindered you to receive a diagnosis?
   1. What do you think would have hindered others to receive a diagnosis?
9. The average time to diagnosis for individuals with PMDD is 9 to 13 years. Why do you think this is so?

**Experiences in the Healthcare System**

“Now we are going to dive deeper into any specific experiences you or others you may know have had within the healthcare system.”

1. What have been your general experiences with patient-doctor communication around the topic of PMDD?
2. Compared to other conditions, have you had better, worse, or the same type of experiences with doctors around PMDD?
   1. How so?
   2. Why do you think that is?
3. Have you ever felt listened to or “heard” by doctors when discussing PMDD?
   1. Have not felt “heard” when discussing PMDD with providers? Please describe one such experience.
4. PMDD already causes much emotional distress due to its impact on mental health and hormonal imbalance. How do you think the typical experience of PMDD in the healthcare system impacts this type of distress?
5. Have you ever had anyone not believe your symptoms or make you doubt your symptoms?
   1. Have you heard of others experiencing this in the healthcare community as well?
   2. Why do you think this happens? Do you think this happens to other people who do not have PMDD?
6. What have been your positive experiences within the healthcare system related to PMDD?
   1. What have been your negative experiences within the healthcare system?
7. How have your previous experiences within the healthcare system with PMDD impacted later experiences in the healthcare system?
8. What have been your experiences with treatment of PMDD within the healthcare system?

**Suggestions**

“Now we are going to talk about any suggestions you may have for the treatment or diagnosis of future PMDD patients.”

1. In your opinion, what could healthcare providers do to create a better experience with the diagnosis or treatment process with PMDD?
2. If you could do anything different about your experience with diagnosis or treatment, what would you have wanted to be changed?
3. Do you have any other suggestions for how people could receive a diagnosis quickly for PMDD?
4. Do you have any other suggestions for how PMDD could be treated?

“Thank you so much for your time today and for sharing your story with me. Do you have anything else you would like to add before we finish?”

**Closing**

“Please remember that this entire interview and its information will remain confidential. The audio recording will be typed up into a transcript and any identifying information will be removed. If you choose to withdraw your interview from the study, please let me know. If you have any additional questions later, feel free to contact me- you have my email address.”

Interview Notes:

**Appendix B: Codebook**

| **Diagnosis** | | |
| --- | --- | --- |
| Pathways to Diagnosis | This code is applied to any description of the experience the participant had to reach their diagnosis. This should accurately describe what the participant’s journey looked like to gain a diagnosis. Any experience after the diagnosis should be coded under “Post Diagnosis.” Even if the participant has self-diagnosed but hadn’t received an official diagnosis yet, code their experience until diagnosis under this category. Double code this with “Post Diagnosis” if the participant’s story overlaps with a mention of how they felt after they were diagnosed (for context). Situations even after diagnosis until finding the right provider should also apply in this code. This should only apply to lived experiences. When asked how many doctors they have been seen for PMDD, apply this code. | *P: So, I umm... was back n the fall of... I’m trying to think what year, I think it was 2018, umm... and I just like started to have umm...a... like a lot of depression umm and anxiety and I immediately linked it to my cycle because I’ve had issues with severe pain for a long time and I’ve always had like hormonal acne and just different things, so different hormonal problems, and I very quickly linked that, like depression and anxiety, to umm my uhh menstrual cycle. So it got to be like pretty bad, pretty debilitating, and I quickly diagnosed myself, via, you know internet searches, and I was like confident that’s what I had ‘cause it matched exactly. So that was kinda how I first found out about it.* |
| Misdiagnosis | This code should directly apply to any experiences relating to misdiagnosis of PMDD. If the participant states that they had a previous diagnosis before PMDD or a doctor almost misdiagnosed them with a different disorder, apply this code. This can be hypothetical or lived experiences. | *That was the first time because, because previously all of my symptoms had been assigned to bipolar um.. And I will say that um.. [sigh] so yeah, so really I never even brought it up to my gynecologist except to tell them the meds that I was on. And I've used hormonal birth control since college*. |
| Post Diagnosis | This code applies to any feelings or change of perception after they received a diagnosis. The participant’s experience after their diagnosis, whether they felt validated or understood why they suffered with PMDD symptoms years before their diagnosis. Any acceptance of the condition must also be coded with this code. This should only apply to lived experiences. Any grieving over lost years after their diagnosis is included in this code. Apply this code if the participant mentions years lost after being misdiagnosed or not being diagnosed for so long. This can apply to any type of “PTSD” the patient may have when going to doctors after having bad experiences with previous ones. | *I didn’t have health insurance and I was spending a thousand dollars a month on meds. A thousand dollars! Like are you kidding me. And then the other piece that’s been really frustrating I have- had all of these like [air quotes] chronic pain issues. But they flare up once a month! And of course- and I kick myself now for not seeing these sort of patterns but I've probably spent ten thousand dollars on physical therapy, massage, acupuncture like you name it. Trying to- and seeing I've had cat scans. Ive had MRIs. I’ve seen surgeons. To try and figure out how to cure this back pain. And now that I'm treating PMDD. I have no back pain.* |
| Designation of Diagnosis | Any discussion of how PMDD should be diagnosed, who should diagnose it, and any difficulties that current providers have with designating diagnosis or care of PMDD should apply to this code. This code applies to the label of PMDD as a diagnosis, PMDD in the DSM, or any types of difficulties in diagnosis such as lack of testing available. If the participant mentions type of provider (OBGYN, Psychiatrist) but only in a hypothetical context of who should treat or diagnose, only code that statement under this code and do NOT code under “OBGYN” or other specialty code. Any problems with current methods of diagnosis should be applied here. This can apply to hypothetical or lived experiences. | *And what I asked for was for them to have empathy you know really think about it from our perspective where many of us have gone through a lot of our life with this disorder without knowing what it was, with being misdiagnosed, with being you know put in this loop in the medical system where your gynecologist tells you to go see a psychologist and your psychologist tells you to go see a gynecologist who then tells you to see and endocrinologist, and you're just lost in this loop because nobody really knows how to treat it because it is such a multi system disorder..* |
| **Specialty** | | |
| OBGYN | Any mention of OBGYN or Gynecologist, including interactions or experiences should be applied to this code. Apply this code even if the participant refers to the doctor with a pronoun such as “she/ he/ they did this…” Do not apply this code to hypothetical situations. | *It’s easier to talk to OB/GYN’s about cycle related issues because it comes up, and just like a standard, um, primary care provider setting they say ‘Okay, when was your last period?’, and that was, and that's usually it for that line of questioning. Um, they are not asking ‘Well, are you having painful periods?, or are you having any of these other symptoms?’ Um, they don’t really go into... asking about the menstrual cycle beyond, ‘are you having a period?, are you pregnant?’ unless you bring it up very intentionally. So, I think it’s a little easier to work it into... um, an OB/GYN visit because they tend to ask more of those in-depth questions related to the menstrual cycle.* |
| Endocrinology | Any mention of Endocrinologist, Reproductive Endocrinologist, etc - including interactions or experiences should be applied to this code. Apply this code even if the participant refers to the doctor with a pronoun such as “she/ he/ they did this…”  Do not apply this code to hypothetical situations. | *It was always a struggle there, so I don’t know, this is kinda indirectly answering your question, but the like my hormones would look fine, umm, and so the OBGYNs and endocrinologists and all like they would just feel it’s not their field.* |
| General Practitioner | Any mention of General Practitioner, including interactions or experiences should be applied to this code. Any Family Practice Doctor, Primary Care Physician, Pediatrician, or Physician’s Assistant should be included. Apply this code even if the participant refers to the doctor with a pronoun such as “she/ he/ they did this…” Apply this code to other mentions of general doctors also. This code can apply to hypothetical or lived experiences. | *P: My current umm physician’s assistant. Umm Who has drawn y’know multiple blood panels, has helped... umm... y’know she put me on like 10 thousand of vitamin D. Which really helps some things. It just like.... all those little things that most providers would skip over. That was really helpful for me. Umm, and she was working in conjunction with my psychotherapist, and that was, like that was it for me.* |
| Mental Health | Any mention of Mental Health Practitioner, including interactions or experiences should be applied to this code. Any instances of Psychiatry, therapist, counselor, or psychotherapist should be included. Apply this code for experiences in mental health institutions as well. Apply this code even if the participant refers to the doctor with a pronoun such as “she/ he/ they did this…” Apply this code if the interviewee mentions PMDD as a mental health condition or they were told by doctors that is psychological. This can be hypothetical or lived experiences. Apply this code to any mental health related stigma as well. SSRIs or any mental health treatments should not be included here but instead under “Treatment.” | *The problem with psychiatrists is that they only know medications. They only know medications. They know nothing else like most psychiatrists don’t even do talk therapy like all they do is see you for 15 minutes and you say, I like my meds I don’t like my meds and they fix your meds like that’s all they freaking do so their worthless in the treatment PMDD from my perspective.* |
| Negative Interactions | This code should apply to any negative interactions with providers. Any instances of doctors not believing them, pushing treatment off to someone else, invalidating the patient, stating that a condition is normal when it is in fact not normal, and forcing the patient to prove themselves to the doctor should be applied. This code should also describe any negative instances with providers that impact later experiences (trauma). Apply this to both lived and hypothetical situations. | *Um, they would [chuckles] just blow me off and... tell me I was a hypochondriac, which I'm not [slight offensive tone]. I, I legitimately have an alphabet soup of stuff that takes a lot of effort to manage so... I, I think I may have... put some of those fears that were instilled in me by my parents on to talking to those providers.* |
| Positive Interactions | This code should apply to any positive interaction with providers. Any hypothetical situations in which participants would like to be heard by providers should be applied here. | *My current umm physician’s assistant. Umm Who has drawn y’know multiple blood panels, has helped... umm... y’know she put me on like 10 thousand of vitamin D. Which really helps some things. It just like.... all those little things that most providers would skip over. That was really helpful for me. Umm, and she was working in conjunction with my psychotherapist, and that was, like that was it for me.* |
| **Coping** | | |
| Self Advocacy | This code applies to any instance of the participant having to advocate for themselves in the healthcare system. Apply this code when a patient must figure out their own health, fight for their diagnosis, and research their treatments on their own. Apply this code to descriptions of self-diagnosis. | *I: Right. And how did it feel to, y’know, almost diagnosis yourself, and you have to be confident in your own diagnosis and confident, you have to find your own providers, and almost find your own treatments, I mean, you’re having to be your own provider pretty much.*  *P: Yes! Yes. It, yeah. Umm, it is really an awful experience, umm, and it is extremely stressful. Umm, you really are, yes, your own provider. Umm, and then you know, you’d have no more than two appointments with the doctor and then become extremely discouraged and move to the next one, and just, yeah, umm, that also I think increases the severity of the symptoms. Because your stress- your stress levels are so high.* |
| Treatment | Apply this code to any type of treatment that the participant has tried in the past, is currently on, or has considered. This can apply to any type of treatment, whether they have tried or not. Apply this to any testing as well (as this is a pathway to treatment). | *P: umm, again, she’s never mentioned it, even though I would, I would like describe exactly, y’know, to her. Cause for me it was like, day 13 or 14, umm all the way to day 5, so you know I’d just get maybe a week, if I was lucky, of relief. Umm But it was, yeah, it was terrible. Umm and again, it was just, it was just said to be normal. Umm and So it was very invalidating, umm... and very much just stick you on a umm the run-of-the-mill treatment which is standard birth control. Whatever formula, y’know, whichever formula is covered by your insurance that’s cheapest. Like that kind of thing, so-* |
| Non Traditional | Apply this code for any type of non traditional providers, treatments, or methods of healthcare. This can refer to functional doctors, holistic treatments, supplements, yoga, and non western medical treatments. This can also include non traditional therapies such as RTT (double code under “Mental Health.”) Double code this under “Treatment” if the patient has actually tried or is currently on this type of treatment. | *P: Yeah, I think a lot of people have had to find alternate sources, um something that I'm also a huge advocate for psilocybin. I started doing a regular regimen, where I would take under 1/2 gram, I believe, I mean it's the tiniest amount, um every four days and you're not experiencing the psychedelic effects that people think of or talk about when they talk about like a mushroom trip. When you're taking a micro-dose…the noise in my head stops, and I noticed that it lasts for day, I'll go days without having that constant rattle in my head, um I feel more connected with everything, I just feel more connected with nature, I see so much more beauty in things and the depression almost disappears.* |
| **Barriers** | | |
| Impact of PMDD | This code applies to descriptions of symptomology and the impact of PMDD on the individuals’ or other PMDD suffers’ lives. This should describe the individual experience of PMDD. If the patient experience includes provider interactions, include this under the codes of “Specialty” instead. This can be positive or negative influences of PMDD on their life. This code can apply to lived or hypothetical situations. | *You know whether that be because of the different sensory issues and emotional issues that it makes thriving in society difficult, or because you're simply trying to preserve your relationships and avoid those moments of rage, or you know, not feeling comfortable in your emotions, not feeling comfortable in your body [chuckles], um you know, the the body aches, the migraines, the the joint pains.* |
| Access to Treatment | This code applies to any type of barriers to access treatment such as cost, availability, and lack of support. If Covid has impacted access to treatments, code this only under “COVID Influence” instead. This can be perceived or lived discussions or experiences. Apply this to access to care or providers as well. | *But I also don’t think that individuals turning to eastern medicine is all- is the answer either right because most people don’t have scientific training and so they assume that if it works for them, this is what I should tell everyone to do as opposed to having someone actually guide you through a process that is at least informed by more than one person’s experience um so I- and- and so for me I continue to be pissed off about this right because I spend $600 a month right now to see this person right I- she charges me $400 to walk into her office and I’m spending about 200$ a month on supplements and that is not sustainable and that is 100% out of reach for most people.* |
| Unawareness | This code applies to any lack of education, ignorance, or unawareness about PMDD. If any participant mentions the lack of understanding for the condition, apply this code. | *Um, and with PMDD they just ignore it because they don’t know what to do with it. They don’t process it like doesn’t fit anywhere so they just like [throwing hand motion] I don’t have a spot for you on my wall so I'm going to discard you.* |
| PMS vs PMDD | This code applies to any discussion of PMS versus PMDD, whether the differences between them or doctors mentioning PMS instead of PMDD as a cause of PMDD symptoms. | *I think PMS is... more of something that—it’s kind of like, um, the difference between, like, baby blues and postpartum depression, you know? Like, it’s definitely more severe and you can’t just, like, turn it off. It lasts longer, I feel like, and there’s, um, more—more symptoms that go along with it.* |
| Misogyny | When a participant describes any type of sexism or role as a woman in society that may impact PMDD or diagnosis and treatment for PMDD, apply this code. If a provider mentions that someone is overemotional because she is a woman, include this code. Apply this code for any menstrual related stigma or women’s health related stigma. Any type of description of misogyny should apply as well. Apply for lived or hypothetical situations. | *There's part of that cultural conditioning that, ‘No no, put everyone else first. You- you- you do the bare minimum for yourself. Take care of everyone else.’ And it’s really hard, um... to overcome that conditioning with time, with resources, um... and even like giving yourself the time and attention you need to take care of yourself. It’s impossible to take care of anyone else if you’re falling apart.* |
| **Suffering** | | |
| Suicidal | Any time a participant mentions suicidal ideation, suicide attempts, or risk of suicide from PMDD, apply this code. Apply this code even for hypothetical situations. | *And it’s the same thing with PMDD. The... suicide risk is so high but, you know, because they don’t... think that they themselves only them can treat it, then they’re like ‘Oh okay, I’ll just refer you to someone else’, but you’re referring someone who is high risk of death.* |
| **Future Recommendations** | | |
| Support for PMDD | Apply this code for any mentions of support needed for PMDD. Apply it also when a participant mentions family, friends, doctor, or PMDD community support they received. Any support they wished they had should also be applied here. Research for PMDD should also be included here. | *Um... but being able to give people their lives back, um, with research and science, that’s the ultimate goal because these people need... help. You cannot do it on your own. It’s just impossible.* |
| Recommendations | Apply this code for any description of recommendations for how PMDD should be diagnosed or treated. This code is also applied for how they wish PMDD would ideally be approached in the healthcare system. This can include recommendations for future PMDD sufferers or doctors as well. | *P: Oh, thank you, no I think um, in I mean in so many things, um I think it should be the case, you know I think that for really extreme illness, yes you do have a care team. Um you have you know, for cancer patients, you have many people in your treatment, right? But with diseases that aren’t as big of a problem, there’s just something lacking, um I think it’s really up to the patient then to piece things together. Which, in some cases, maybe the patient wants to piece things together and have more control over their healthcare, um and some cases, it probably is…you know, is devastating.* |
| **Other Factors** | | |
| Other Condition | This code should be applied for any description of a medical condition other than PMDD. Any premenstrual condition or medical condition that the participant may have, a family member may have, or any hypothetical situation regarding a non PMDD condition should be applied here. | *People know what PCOS is. They understand that. They can look at you and see, ‘Oh yeah! You- you have a lot of hair [chuckles] on your face.’ Or ‘Yeah, I see a hump between your shoulders, and the extra weight, and we know what to do for that. Here’s some birth control, here’s some metformin, here’s some diet advice that will help with your condition. Here you go. Here’s all the resources in the world.’ And then PMDD, they’re like, ‘Uh... we can give you antidepressants, which one do you want to try?’ [laughs] Like, really! Because they hear depressed, upset, crying, anxiety. It’s like, okay, that fits this script we have for anxiety and depression.* |
| COVID Influence | Apply this code when any impact of the COVID-19 Pandemic on anything PMDD related is described in the interview. | *I think it's changing I think it's starting, just even in the past year with COVID and the mental health crisis that we all saw, I think it's opened up everybody's eyes a lot more to all of the issues that we're experiencing, not just in mental health but also in female health.* |
| Childbearing | Apply this code to any mention of child bearing, pregnancy, or fertility in relation to PMDD. For instance, if a participant describes that a doctor only cared about women’s health in relation to child bearing instead of menstrual problems, apply this code. | *But then, it’s... there was a time when umm he basically just said “I can’t help you anymore”, umm and then he said some very invalidating things. Umm, including like, just kind of off the cuff, like well you know you’d have to see if you would even be able to have biological children, and that’s not the kind of thing that you just throw at a 19-year old girl, you know? So, umm, yeah, just kind of weird things like that. Umm, so I- I can’t really trace a complete, like trend in male versus female.* |
